# Seroprevalence and molecular surveillance of *Dirofilaria immitis* in shelter dogs from central New Mexico, USA

**DOI:** 10.1186/s13071-026-07341-0

**Published:** 2026-08-20

**Authors:** Maureen A. Kelly, Alexa Starnes, Mary Schech, Erin Clarke, Rafael A. N. Ramos, Hassan Hakimi, Pabasara Weerarathne, Guilherme G. Verocai

**Affiliations:** 1 Department of Veterinary Pathobiology, College of Veterinary Medicine and Biomedical Sciences, Texas A&M University, College Station, TX 77843 USA; 2 Albuquerque Animal Welfare, Albuquerque, NM 87112 USA; 3 Laboratory of Parasitology, Federal University of the Agreste of Pernambuco, Garanhuns, PE 55292-270 Brazil; 4 Present Address: Department of Diagnostic Medicine and Pathobiology, College of Veterinary Medicine, Kansas State University, Manhattan, KS 66506 USA

**Keywords:** Antigen detection, Canine heartworm disease, *Canis lupus familiaris*, Microfilariae detection, Molecular diagnostics, Probe-based qPCR, Serology

## Abstract

**Background:**

*Dirofilaria immitis*, the causative agent of canine heartworm disease, is a mosquito-borne filarial nematode, endemic to most of North America. Clinical signs may include coughing, exercise intolerance, abnormal heart sounds, and lethargy, which can lead to heart failure and death in severe cases. For accurate diagnosis, the American Heartworm Society (AHS) recommends testing dogs annually for the detection of heartworm antigens and microfilariae. In this study, we assessed the prevalence and potential risk factors for *D. immitis* in dogs from Albuquerque, New Mexico, using antigen and microfilariae detection tests.

**Methods:**

In 2023, serum and whole-blood samples (*n* = 402) were collected from dogs in Albuquerque, New Mexico, as part of a larger study to detect *Onchocerca lupi*. Serum samples were screened for *D. immitis* using a commercially available antigen-detection ELISA (DiroCHEK^®^, Zoetis) with pre- and post-immune complex dissociation (ICD) via heat treatment, although this is not currently recommended by AHS. Following genomic DNA extraction, we also screened whole blood using a newly optimized probe-based real-time polymerase chain reaction (qPCR) assay. To assess the potential association between *D. immitis* presence and risk factors, including age, sex, breed group, and coat color, a univariate analysis was performed using Fisher’s exact test or the chi-squared test.

**Results:**

Serum of 4 (0.99%) dogs tested antigen-positive pre-ICD, while 14 (3.47%) dogs tested antigen-positive post-ICD. We did not detect any positive dogs for *D. immitis* microfilariae by the probe-based qPCR. No risk factors were associated with the detection of the *D. immitis* antigen in this dog population.

**Conclusions:**

Although microfilariae were not detected in this study, it is possible that infections were in the early stages of disease progression, with *D. immitis* detected in pre- and post-ICD via antigen testing. Low seroprevalence and the absence of microfilariae DNA detected highlight the importance of using both pre- and post-ICD antigen detection alongside molecular-based microfilariae tests, particularly for companion animals living in low-endemic regions and for shelter populations that are less likely to be on heartworm prevention.

**Supplementary Information:**

The online version contains supplementary material available at 10.1186/s13071-026-07341-0.

## Background

*Dirofilaria immitis* is a vector-borne filarial nematode found worldwide and the causative agent of canine heartworm disease, with the potential to cause fatal outcomes, making it of great importance in veterinary medicine [1–3]. The life cycle of *D. immitis* is complex and requires a mosquito as an intermediate host, which transmits the infective stage (third-stage larvae) to susceptible definitive hosts, with a prepatent period of 6–7 months [1–4]. Dogs infected with *D. immitis* exhibit a range of clinical signs, from mild signs such as coughing, exercise intolerance, and lethargy to more severe signs, including abnormal heart and lung sounds, hepatomegaly, dyspnea, ascites, and caval syndrome, which can be fatal [1–4]. Due to the severity of the disease and the need for accurate diagnosis, the American Heartworm Society (AHS) recommends a serology-based test and a microfilariae detection test (MFDT) to confirm infection of *D. immitis* [1–5].

Serology-based testing, such as enzyme-linked immunosorbent assays (ELISA), can detect antigens produced by the reproductive tracts of at least three adult female *D. immitis* [6–11]. These commercially available serological assays are widely used in both clinical and research settings; however, despite their demonstrated high diagnostic sensitivity and specificity to date, false-negative results can still occur [12–15]; for example, in infections involving fewer than three females, male-only infections, or early parasitism involving only juvenile adults [10, 16, 17]. There is also a possibility of immune complex formation, which can be disrupted by heat treatment, allowing for the release and detection of antigens [6, 7, 10].

DNA-based techniques for MFDTs, such as conventional PCR (cPCR), real-time PCR (qPCR), and droplet digital PCR (ddPCR), are increasingly available as send-out options from reference laboratories [18–24]. These techniques can aid in confirming *D. immitis* in suspected or inconclusive cases in clinical settings or in large-scale surveys that screen populations at risk, such as dogs, cats, and vectors [18, 23, 24]. Although microscopy-based MFDTs (e.g., modified Knott’s test, blood smear) are more widely used in clinical settings, molecular assays exhibit higher sensitivity and specificity, enabling detection of only *D. immitis* infections and serving as essential tools for large-scale surveillance studies [18, 23, 24].

Currently, the AHS and other organizations (e.g., the Companion Animal Parasite Council (CAPC)) conduct active surveillance using clinical data on *D. immitis* to understand the nationwide impact, generating incidence and prevalence maps [2, 25]. For AHS, each map estimates overall incidence using data collected at 3-year intervals, and the most recent map reports the highest nationwide incidence to date, with the highest number of cases reported in the southeastern USA (Supplementary Data: Fig. S1) [2]. A similar estimated prevalence of *D. immitis* has also been reported in CAPC’s prevalence maps at the county, state, and national levels (Supplementary Data: Fig. S2) [25]. However, for both organizations, relatively fewer cases are reported in the southwestern states, specifically New Mexico, which may be due to lower *D. immitis* endemicity in those states. This could also be due to limited data collection in these areas, finite reporting clinics, a lack of annual testing, or a limited number of established clinics, resulting in an underreported prevalence in these regions [26, 27]. Nonetheless, active surveillance within at-risk populations, such as shelter dogs, is necessary to better understand the epidemiology of this potentially fatal parasite. Therefore, this study aimed to assess the prevalence and potential risk factors for *D. immitis* infection in dogs from Albuquerque, New Mexico, using recommended methods: a serological test and a molecular-based assay.

## Methods

### Sample collection

Between January and September 2023, both serum and whole blood samples (*n* = 402) were collected from dogs residing in a single shelter in Albuquerque, Bernalillo County, New Mexico, as part of a larger epidemiological study to determine the prevalence of *Onchocerca lupi* [28]. Bernalillo County, one of the largest counties in the USA, is home to the city of Albuquerque, with a warm-temperate, semi-desert climate [29–31]. All sample types were collected opportunistically prior to spay or neuter surgery in dogs, estimated to be older than 6 months [28]. Demographics were recorded at the time of sampling, including estimated age, sex, American Kennel Club (AKC) breed class, and coat color [28]. Once received, samples were aliquoted into 500 µL aliquots and stored at −20 °C until processed for antigen and molecular testing.

### Antigen evaluation

Serum samples were evaluated using a commercially available ELISA antigen detection test (DiroCHEK^®^, Zoetis Inc., Kalamazoo, MI, USA) according to the manufacturer’s instructions, with both positive and negative controls included. Each sample was evaluated pre-ICD (no heat treatment) and post-ICD (heat treatment). For heat treatment, 250 µL of serum was incubated at 103 °C for 10 min, then centrifuged at 16,000 RCF for 10 min at room temperature [6, 32]. Both pre- and post-ICD samples were processed and interpreted according to the manufacturer’s protocol (Zoetis Inc., Kalamazoo, MI, USA). Once completed, each sample was assessed for visual color change within 5 min. If a color change occurred after 5 min, the sample was considered positive for *D. immitis* antigen. If a color change was not visible within the allotted time, the samples were recorded as no antigen detected (NAD). Following the initial 5 min, results were also measured using a spectrophotometer (BioTek Synergy H1 Microplate Reader, Agilent, Santa Clara, California, USA) to quantify optical density (OD) at 490 nm [6, 18, 32]. Following quantification, each sample was compared with the OD records of both positive and negative controls. To be considered positive, the recorded OD value had to exceed the negative control value on the corresponding plate.

### DNA extraction and qPCR

Genomic DNA from each whole blood aliquot (500 μL) was extracted using the Maxwell^®^ 48 RSC (Promega Corporation, Madison, Wisconsin, USA) with the Whole Blood DNA Kit (Promega Corporation, Madison, Wisconsin, USA) according to the manufacturer’s instructions. Samples were then screened for *D. immitis* using a previously published probe-based qPCR protocol [18], including a commercially available internal positive control (IPC) (VetMAX^™^ Xeno^™^ Internal Positive Control DNA, Thermo Fisher Scientific Inc., Waltham, Massachusetts, USA). This assay targets a 166 bp fragment of the cytochrome oxidase subunit 1 (*cox1*) region of mitochondrial DNA using primers (Fil.COI.749-F-5′-CATCCTGAGGTTTATGTTATTATTTT-3′; Fil.COI.914-R-5′-CWGTATACATATGATGRCCYCA-3′) with a TaqMan^®^ probe (FAM-5′-TATTTGGATTGCTGTATGGGG-3′-non-fluorescent quencher-MGB). All reactions were performed in a 20 µL reaction containing 1.5 µL of molecular-grade water, 0.5 µL (50 µM) per primer, 0.5 µL (20 µM) of probe, 10 µL 2 × of TaqMan^®^ Fast Advance Master Mix (Applied Biosystems, Waltham, Massachusetts, USA), 1 µL of VetMAX^™^ Xeno^™^ IPC—VIC^™^ Assay (ThermoFisher Scientific Inc., Waltham, Massachusetts, USA), 1 µL of VetMAX^™^ Xeno^™^ IPC (10^–2.5^) (ThermoFisher Scientific Inc., Waltham, Massachusetts, USA), and 5 µL of DNA template. All qPCR reactions were performed on a Quant-Studio 3 real-time PCR system (Applied Biosystems, Waltham, Massachusetts, USA), following standard universal cycling conditions, which included a denaturation stage at 95 °C for 20 s, followed by 40 cycles of a two-step PCR stage at 95 °C for 1 s and 60 °C for 20 s. All singlicate runs also included two positive controls and a no-template control. One positive control consisted of extracted DNA from an adult *D. immitis* specimen, confirmed both morphologically and molecularly. The second positive control used DNA from VetMAX^™™^ Xeno^™^ IPC. For the no-template control, nuclease-free molecular water was used. Results of each qPCR were analyzed using Design & Analysis 2 software to determine the presence or absence of *D. immitis* within each sample (Applied Biosystems, Waltham, Massachusetts, USA).

### Data analysis

Statistical analysis was performed using STATA^®^ version 19.5 BE-Basic Edition (College Station, TX, USA) to determine the association between demographic variables (e.g., age, sex, AKC breed group, and coat color) and the outcome (*D. immitis* presence) for each diagnostic test. A univariate analysis was performed using either a Fisher’s exact or chi-squared test, depending on the number of positives per test, with a *P*-value cutoff of *P* ≤ 0.05.

## Results

**Fig. 1:**
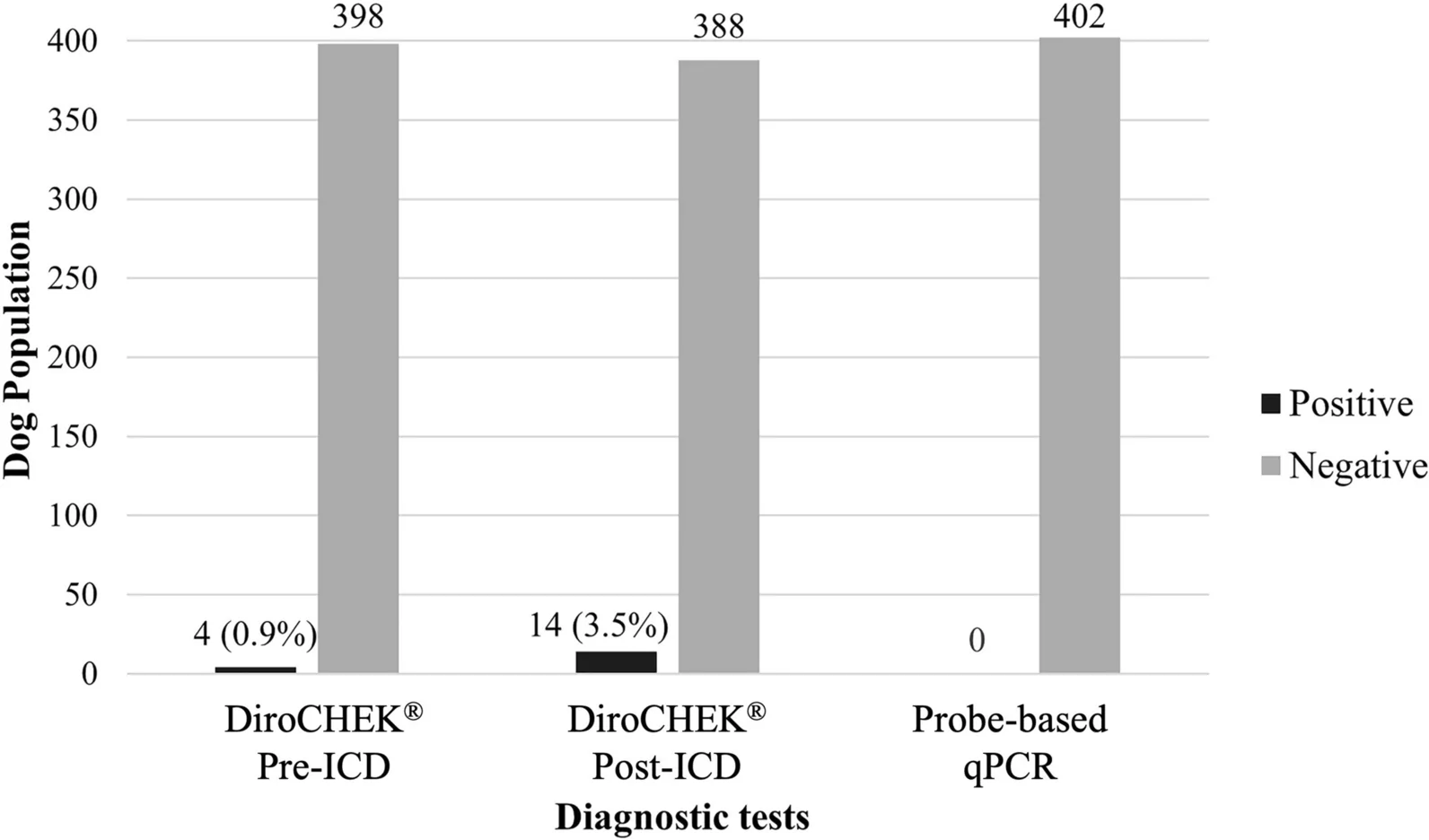
Summary of the results from pre- and post-ICD antigen detection and probe-based qPCR for *Dirofilaria immitis*

The overall results of all diagnostic tests including antigen testing pre- and post-ICD, as well as probe-based qPCR, are summarized in Fig. 1. Positivity rates of 0.99% (*n* = 4/402; 95% confidence interval (CI) 0.27–2.52%) and 3.47% (*n* = 14/402; 95% CI 1.91–5.77%) were observed for pre-ICD and post-ICD, respectively. Table 1 presents all positive OD values for each dog, including both positive and negative control values. All samples were assessed by visual inspection, and OD values were compared with the positive control. All samples positive via pre-ICD (*n* = 4/402) exhibited color change; however, two did not match or exceed the positive control OD value (Table 1). Samples that were positive via post-ICD (*n* = 14/402) showed a visual change in color, but 11 samples did not have a high OD value when compared with the positive control (Table 1). No dogs were positive for *D. immitis* microfilariae DNA via the probe-based qPCR. The univariate analysis did not reveal any potential risk factors (i.e., age, sex, AKC breed group, and coat color) associated with *D. immitis* antigen detection (Table 2).

**Table 1:** Summary of the visual color change and optical density (OD) values of DiroCHEK^®^ reported for all antigen-positive dogs from Albuquerque, New Mexico (*n* = 402)

Sample no.	Visual color change	OD pre-ICD	Visual color change	OD post-ICD	Visual color change	Positive control OD	Visual color change	Negative control OD
39	Y	0.134	Y	0.183	Y	0.104	N	0.053
136*	N	–	Y	0.067	Y	0.135	N	0.056
168	Y	0.105	Y	0.122	Y	0.110	N	0.056
174	N	–	Y	0.053	Y	0.077	N	0.049
189	N	–	Y	0.053	Y	0.077	N	0.049
275	Y	0.048	Y	0.072	Y	0.071	N	0.046
291	N	–	Y	0.049	Y	0.061	N	0.045
292	Y	0.055	Y	0.060	Y	0.061	N	0.045
300	N	–	Y	0.049	Y	0.061	N	0.045
309*	N	–	Y	0.058	Y	0.138	N	0.055
341^†^	N	–	Y	0.074	Y	0.136	N	0.060
343^†^	N	–	Y	0.062	Y	0.136	N	0.060
354	N	–	Y	0.047	Y	0.056	N	0.045
371	N	–	Y	0.051	Y	0.063	N	0.047

*Cercopithifilaria bainae co-infection; ^†^*Onchocerca lupi* co-infection, as per Kelly et al. [28]

**Table 2:** Summary of demographic characteristics of the sampled dog population from Albuquerque, New Mexico (*n* = 402)

Variable	Total no.	*Dirofilaria immitis pre-ICD positive*			*Dirofilaria immitis post-ICD positive*		
		No. (%)	95% CI	Fisher’s exact	No. (%)	95% CI	Chi-squared *df* *P*-value
Age							
Juvenile (≤ 1 year old)	120	1 (0.8)	0.0–4.6	1.000	6 (5.0)	1.9–10.6	1.771 2 0.412
Adult (> 1–7 years old)	259	3 (1.2)	0.2–3.4		8 (3.1)	0.4–6.1	
Senior (> 7 years old)	23	0	–		0	–	
Sex							
Female	193	2 (1.0)	0.1–3.7	0.658*	10 (5.2)	2.5–9.3	3.187 1 0.074
Male	209	2 (1.0)	0.1–3.4		4 (1.9)	0.5–4.8	
American Kennel Club Breed Group							
Herding group	88	1 (1.1)	0.0–6.2	0.365	3 (3.4)	0.7–9.6	6.066 8 0.640
Hound group	16	0	–		0	–	
Non-sporting group	32	0	–		1 (3.1)	0.0–16.2	
Sporting group	18	1 (5.6)	0.1–27.3		2 (11.1)	1.4–34.7	
Terrier group	101	0	–		2 (2.0)	0.2–7.0	
Toy group	92	1 (1.1)	0.0–5.9		5 (5.4)	1.8–12.2	
Working group	41	1 (2.4)	0.0–12.9		1 (2.4)	0.0–12.9	
Miscellaneous	12	0	–		0	–	
Unknown	2	0	–		0	–	
Coat color							
Single color	132	1 (0.8)	0.0–4.1	0.601*	4 (3.0)	0.8–7.6	0.119 1 0.729
Multiple colors	270	3 (1.1)	0.2–3.1		10 (3.7)	1.8–6.7	

*One-sided Fisher’s exact; *df* degrees of freedom, *CI* confidence interval

## Discussion

In this study, low seroprevalence (*n* = 4/402; 0.99%; *n* = 14/402; 3.47%) of *D. immitis* was observed among shelter dogs in Bernalillo County, New Mexico. Although considered low when compared with other highly endemic regions of the USA, it is nearly six times higher than what is currently reported by CAPC in Bernalillo County (2023: 0.46%; 2024: 0.54%; 2025: 0.40%) and nearly four times higher at the state level for New Mexico (2023: 0.75%; 2024: 0.82%; 2025: 0.87%) [25]. The positivity reported by CAPC likely results from client-owned dogs receiving consistent veterinary care and monthly chemoprophylaxis, as data reported by various veterinary diagnostic companies [25]. The increase in overall positive dogs observed by CAPC between 2023 and 2025 in both Bernalillo County and New Mexico may be due to a variety of factors, such as more clinics requesting and performing *D. immitis* diagnostics, which can lead to an increased rate, especially in communities that previously had low prevalence (e.g., New Mexico) [25]. Additionally, the possibility exists that rescreening of dogs previously confirmed positive for *D. immitis* that remain positive can affect the overall frequency reported, as these cases are not necessarily newly confirmed cases [1, 2, 25].

In large-scale epidemiological studies, shelter populations are commonly used to estimate the baseline prevalence of vector-borne parasites, including *D. immitis*, and play a significant role in local transmission by acting as reservoirs for vectors [1, 2, 9, 11]. However, the lack of clinical history for dog populations in shelters poses challenges for epidemiological surveys, particularly when animals are translocated in response to natural disasters, import/export, or adoption events [1, 2, 9, 23, 26, 27, 33, 34]. This was a limitation of this study, as clinical history was unavailable at the time of sampling [28]. The movement of heartworm-positive dogs from highly prevalent areas to nonendemic regions has been previously documented [26, 27]. For instance, in the state of Colorado, the effects of importing heartworm-positive dogs contributed to the increase of seroprevalence from 0.5% in 2013 to 0.84% in 2017 [27]. These dogs may also come from areas of the state with little epidemiological data available and/or suboptimal access to veterinary care, including Indian reservations. Although increased prevalence has been observed in CAPC data and in AHS-reported incidence rates in New Mexico, animal translocation, especially in shelter populations, must be further evaluated in future investigations [2, 25].

Of the 14 dogs identified as positive in the present study, 4 were positive in both the pre-ICD and post-ICD samples. Although we cannot correlate parasite burden with the ELISA kit’s color intensity, it is worth noting that all four dogs showed higher OD values post-ICD than pre-ICD (Table 1) [1, 2, 16, 35]. Despite the high sensitivity of the antigen testing for diagnosing *D. immitis*, cross-reactivity can occur with other parasites, including *Dirofilaria repens*, *Angiostrongylus vasorum*, and *Spirocerca lupi*, when heat treatment is implemented [17, 36–39]. It is possible that the dogs that were positive only post-ICD (*n* = 10/14) were false positives, as no dogs were positive by qPCR. To mitigate the potential effects of cross-reactivity, it can be beneficial to use molecular-based techniques in combination with microscopy-based approaches to ensure accurate results, though this may be more practical for research studies than for clinical practice.

It should also be noted that these samples were collected as part of a previously published study focusing on the detection of *O. lupi* [28]. Two dogs (i.e., dogs 341 and 343) that were positive for *D. immitis* via post-ICD testing were also positive for *O. lupi.* This co-infection is likely, as *O. lupi* is a zoonotic filarial nematode endemic to the southwestern USA [28, 40]. It is unlikely that this was a false positive, as *O. lupi* does not cross-react in commonly used antigen tests [41]. In addition to *O. lupi*, the original study reported that 20 dogs were positive for *Cercopithifilaria bainae* (data not shown), a skin-dwelling filarial nematode transmitted by *Rhipicephalus sanguineus* [28, 42]; in the present study, two additional dogs were also positive for *D. immitis* by post-ICD testing (i.e., dogs 136 and 309). *Cercopithifilaria bainae* has not yet been reported to cross-react with commercially available antigen tests for *D. immitis*, but additional data are necessary to refute this possibility. However, the likelihood decreases, given that the remaining *C. bainae*-positive dogs (*n* = 18) did not yield a positive result pre- or post-ICD in this study, suggesting that false-positive results are less likely. Co-infections with blood- and cutaneous-borne filarial nematodes have been documented and considered in large-scale studies, especially in populations with unknown parasitic infections, such as domestic dogs and wild canids [10, 28, 42].

In this study, we did not detect *D. immitis*-positive dogs by probe-based qPCR, with nearly 1% (*n* = 4/402) positive pre-ICD and 4% (*n* = 14/402) positive post-ICD. Several possibilities could explain these discordant results. One possible cause could be that these dogs were examples of “occult infections,” where adult worms are present in the host with an absence of microfilariae circulating throughout the infected animal [2, 7, 10, 43, 44]. There are several examples of occult infections, such as the presence of juvenile adults that produce a detectable antigen but do not produce a significant number of microfilariae [2, 7, 10, 43, 44]. Another possibility is that mature adults are present but too few to reproduce microfilariae [42]. These occult infections suggest the detection was too early to confirm via MFDT. Additionally, given an unknown clinical history, there is a slight possibility that these positive dogs were recently treated with heartworm prevention, which cleared microfilariae from the circulation but did not treat the adult infection [2, 16, 21, 43]. It is also possible that dogs were not microfilariae-positive by qPCR due to low microfilarial concentrations, given the small volume of the aliquot used for DNA extraction, which could have resulted in insufficiently isolated DNA. Nonetheless, in cases similar to this study, it is ideal to perform testing, such as diagnostic imaging (e.g., radiograph, ultrasound), or to retest in 3 months, as recommended by AHS, on these specific dogs to ensure an accurate diagnosis of *D. immitis* [2].

Although we did not identify any significant risk factors associated with *D. immitis*, these results should be interpreted with caution and require additional epidemiological surveys in regions with reported low prevalence (e.g., southwestern USA) [2, 25–27]. This is the first epidemiological study to assess the prevalence of *D. immitis* in shelter dogs in Albuquerque, New Mexico, and reports a nearly sixfold higher prevalence (3.47%) than that reported in nationwide surveillance (0.40%) [2, 25–27]. To further evaluate the increased prevalence of *D. immitis*, assessment of both environmental (i.e., mosquito species presence, temperature fluctuations) and societal factors (i.e., veterinary care availability, access to preventive measures) in highly endemic areas, such as the southeastern USA, and emerging endemic regions, such as New Mexico, are needed in real time [1–25, 44–46]. Within Bernalillo County, mosquito surveillance studies have reported several species with vectorial competence for transmitting *D. immitis*, including *Aedes vexans* and *Culex quinquefasciatus* [47]. Additionally, *Aedes aegypti* was documented in the study area, although it is not considered a vector; and *D. immitis* has been reported in bloodmeal analyses of *A. aegypti* in South Texas, suggesting potential for transmission [48, 49]. These future assessments will facilitate the development of control and prevention strategies of this fatal zoonotic filarial nematode.

## Conclusion

Although microfilariae were not detected in this study, *D. Immitis* antigen was identified through pre- and post-ICD testing, indicating the potential for early infection. The seroprevalence observed in this study was nearly six times higher than that reported by surveillance organizations, such as CAPC, over the past three years [25]. Furthermore, the absence of microfilariae DNA emphasizes the importance of employing both pre- and post-ICD antigen testing alongside molecular microfilariae diagnostics, particularly for companion animals residing in low-endemic areas and shelter animals that may not be receiving heartworm prevention. 

## Supplementary Information

Additional file 1.

## Data Availability

All data supporting the main conclusion of this study are included within the manuscript and provided as supplementary material.
