# Supplementary material for: Seroprevalence and molecular surveillance of Dirofilaria immitis in shelter dogs from central New Mexico, USA
Source: Parasit Vectors. 2026 Aug 20;19:350. doi: 10.1186/s13071-026-07341-0 (PMC13491768; doi:10.1186/s13071-026-07341-0)
Supplement: Supplementary file 1 [file 13071_2026_7341_MOESM1_ESM.docx]

**Supplementary Data, Figure S1.** An incidence map depicting the average number of *Dirofilaria immitis* positive cases per clinic using data collected from veterinary practices across the United States of America. Retrieved from the American Heartworm Society, 2025.

**Supplementary Data, Figure S2.** A prevalence depicting the estimated prevalence of *Dirofilaria immitis* using data collected by multiple veterinary diagnostic companies. Retrieved from the Companion Animal Parasite Council, 2025.

**Supplementary Files, Table S1.** Summary of demographic characteristics of the sampled dog population from Albuquerque, New Mexico (n=402).

| Demographics | No. (%) | |
| --- | --- | --- |
| Age | | |
| Juvenile  (≤1 year old) | 120 (29.9) | |
| Adult  (>1– 7 years old) | 259 (64.4) | |
| Senior  (>7 years old) | 23 (5.7) | |
| Sex | | |
| Female | 193 (48.0) | |
| Male | 209 (52.0) | |
| AKC Breed groups | | |
| Herding group | | 88 (21.9) |
| Hound group | | 16 (4.0) |
| Non-sporting group | | 32 (8.0) |
| Sporting group | | 18 (4.5) |
| Terrier group | | 101 (25.1) |
| Toy group | | 92 (22.9) |
| Working group | | 41 (10.2) |
| Miscellaneous | | 12 (3.0) |
| Unknown | | 2 (0.5) |
| Coat Color | | |
| Single | | 132 (32.8) |
| Mixed | | 270 (67.2) |
